# Supplementary material for: Nanoscale coordination polymers induce immunogenic cell death by amplifying radiation therapy mediated oxidative stress
Source: Nat Commun. 2021 Jan 8;12:145. doi: 10.1038/s41467-020-20243-8 (PMC7794559; doi:10.1038/s41467-020-20243-8)
Supplement: Supplementary file 1 — Supplementary Information [file 41467_2020_20243_MOESM1_ESM.pdf]

Supplementary Information

Nanoscale coordination polymers induce immunogenic cell death by amplifying radiation therapy mediated oxidative stress

Zhusheng Huang[1], Yuxiang Wang[1], Dan Yao[1], Jinhui Wu[1,2,3], Yiqiao Hu[1,2,3,*] and Ahu Yuan[1,2,3,*]

Affiliations:

[1] State Key Laboratory of Pharmaceutical Biotechnology, School of Life Science and Medical School, Nanjing University, Nanjing 210093, China.

[2] Jiangsu Key Laboratory for Nano Technology, Nanjing University, Nanjing 210093, China.

[3] Institute of Drug R&D, Medical School of Nanjing University, Nanjing 210093, China.

These authors contributed equally: Zhusheng Huang, Yuxiang Wang

These authors jointly supervised this work: Yiqiao Hu, Ahu Yuan

Yiqiao Hu, Ph.D. Professor
Address: 22 Hankou Road, Nanjing 210093, China
Phone: +86-25-83596143;
E-mail: huyiqiao@nju.edu.cn

Ahu Yuan, Ph.D. Associate Professor
Address: 22 Hankou Road, Nanjing 210093, China
Phone: +86-25-83596143;
E-mail: yuannju@nju.edu.cn

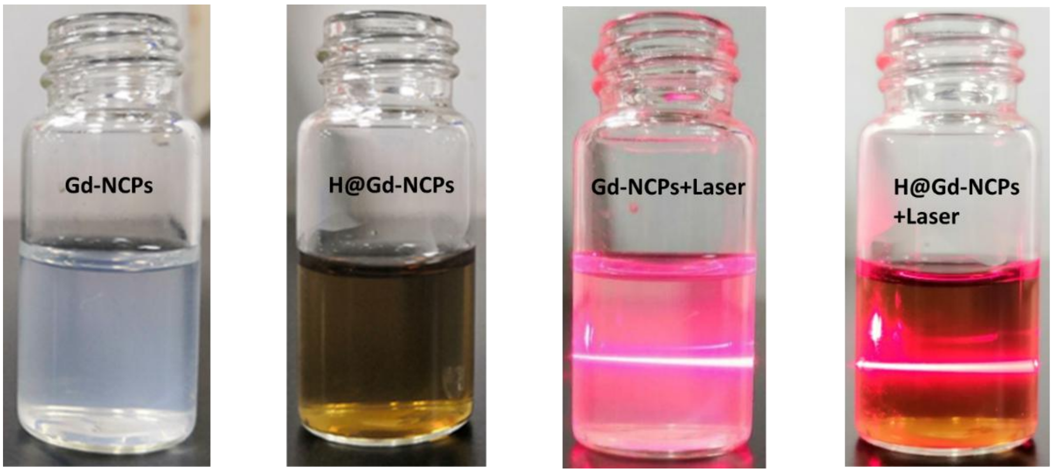

Supplementary Figure 1. Photographs and the Tyndall effect of Gd-NCPs, H@Gd-NCPs.

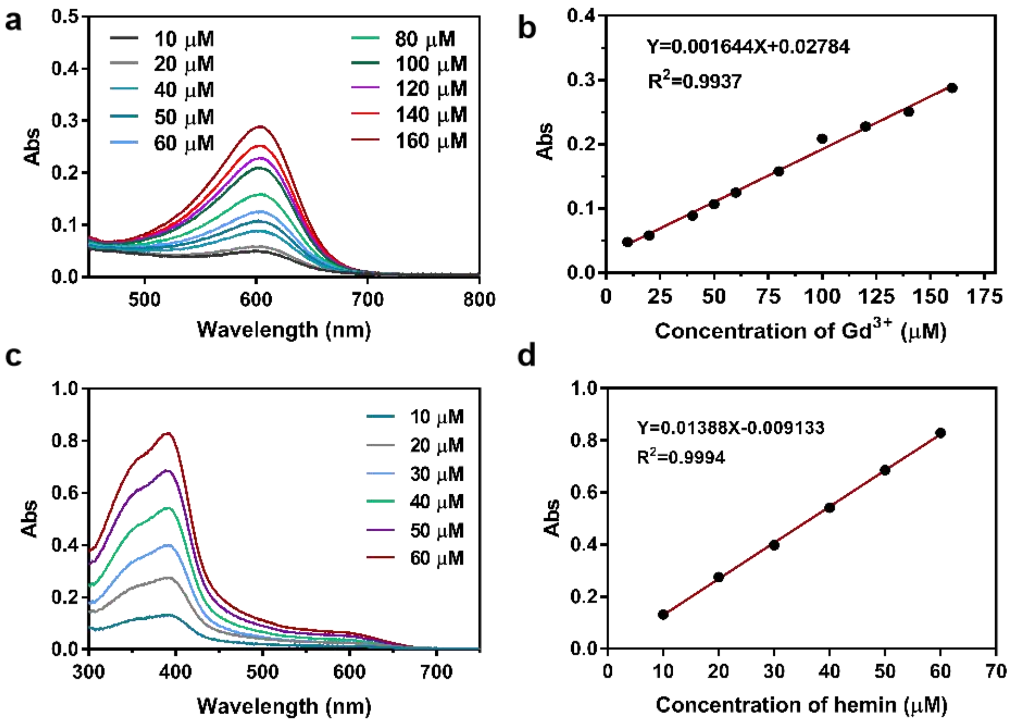

Supplementary Figure 2. Quantitative standard curve of Gd^{3+} and Hemin. **(a-b)** The concentration of Gd^{3+} was determined by adding TC solution. Then, from the absorbance at 605 nm, the quantitative standard curve of Gd^{3+} was obtained. **(c-d)** The calibration curve was obtained by plotting absorbance of Hemin aqueous solution at 392 nm by ultraviolet spectrophotometer. These experiments were repeated twice independently with similar results. Source data are provided as a Source data file.

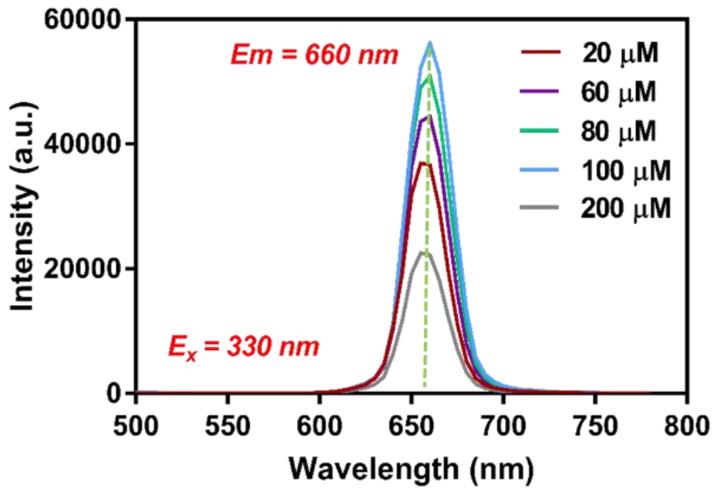

Supplementary Figure 3. Emission spectra of various gradient concentration H@Gd-NCPs with 330 nm excitation at pH 7.4. This experiment was repeated twice independently with similar results. Source data are provided as a Source data file.

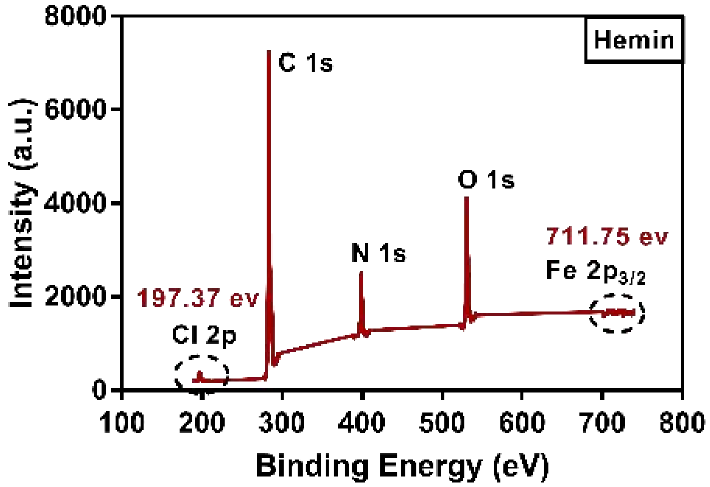

Supplementary Figure 4. Qualitative element analysis of Hemin by X-ray photoelectron spectroscopy (XPS). This experiment was repeated twice independently with similar results. Source data are provided as a Source data file.

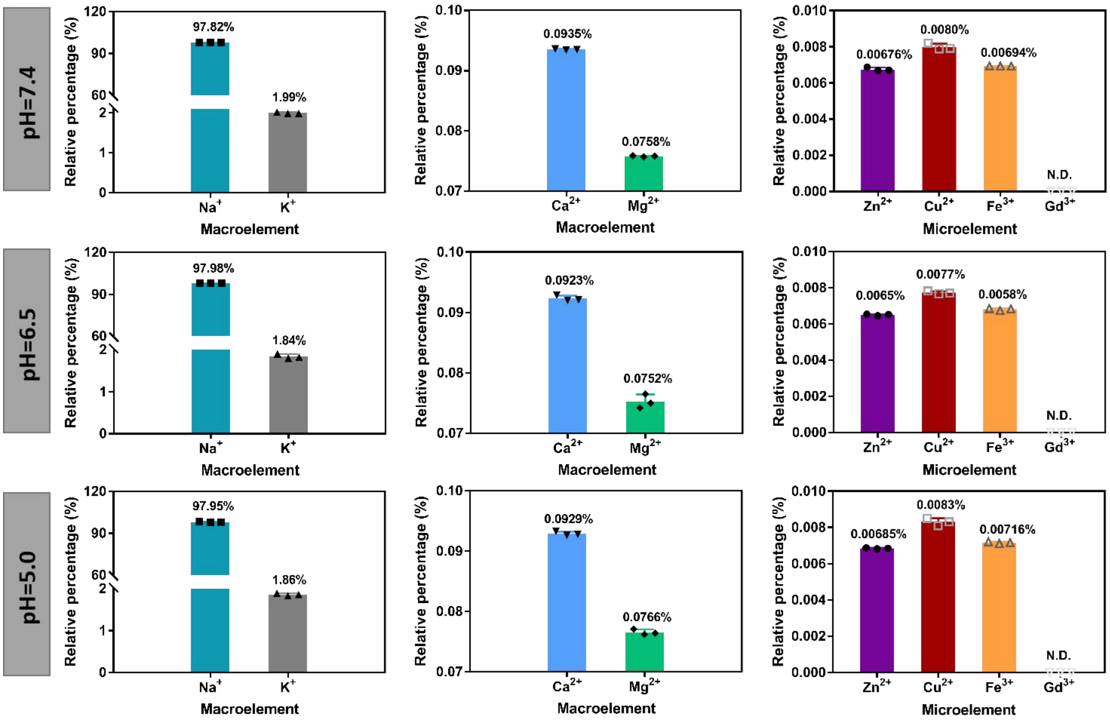

Supplementary Figure 5. Trans-metallation experiments of H@Gd-NCPs under pH = 7.4, 6.5, 5.0, respectively. Analysis of metal ion content in trans-metallation dialysate (50% bovine serum, adding extra [Na^+] = 150 mM, [K^+] = 5.0 mM, [Ca^{2+}] = 2.5 mM, [Mg^{2+}] = 1.25 mM, [Zn^{2+}] = 30 μM, [Fe^{3+}] = 30 μM, [Cu^{2+}] = 30 μM to mimic physiological environment) via ICP-OES (n = 3 biologically independent samples). This experiment was repeated twice independently with similar results. All data were presented as mean ± SD. Source data are provided as a Source data file.

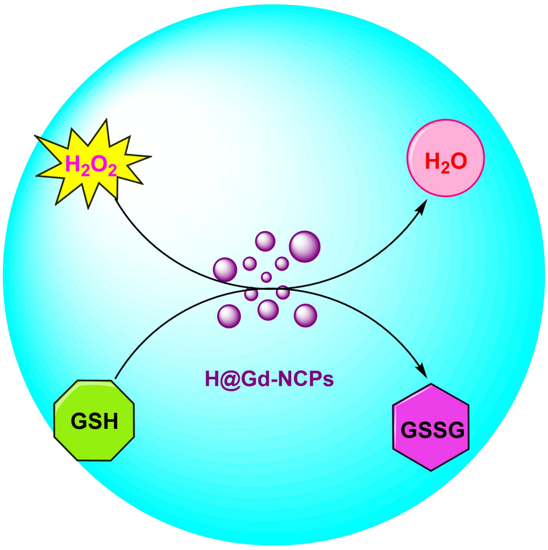

Supplementary Figure 6. Schematic diagram and detailed mechanism of H@Gd-NCPs converts the H_2O_2 and glutathione (GSH) to H_2O and oxidized glutathione (GSSG).

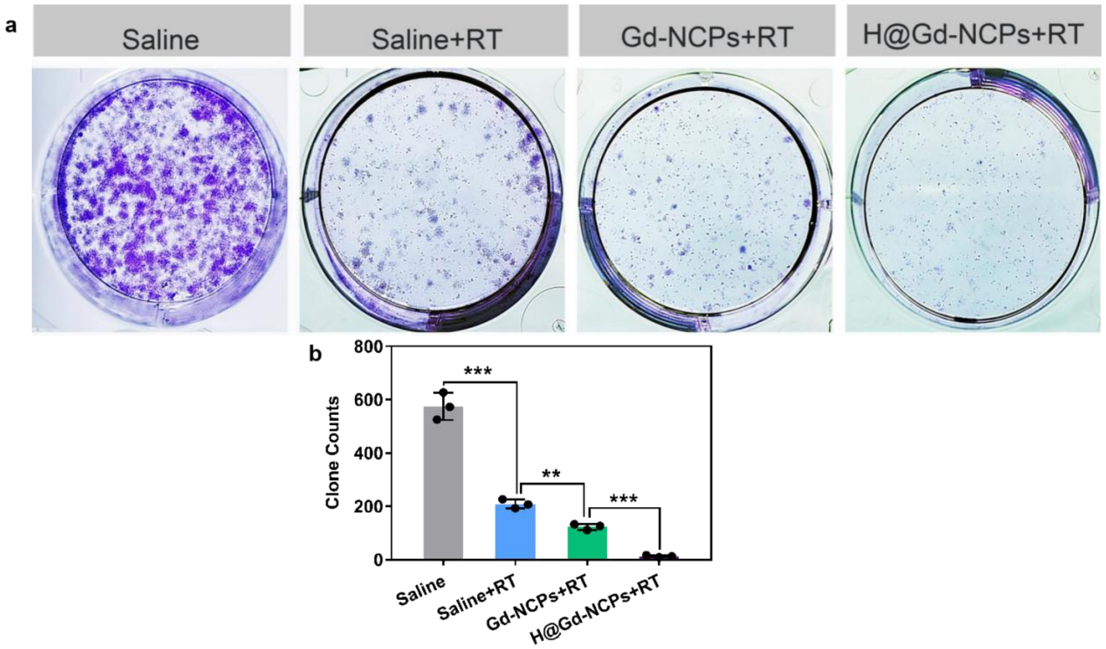

Supplementary Figure 7. Cell clonogenic assay. (a) Images and (b) quantification of CT26 cell clones (n = 3 biologically independent cells, ***p = 0.0003, **p = 0.0017, ***p = 0.0001). This experiment was repeated twice independently with similar results. All data were presented as mean ± SD. Two-sided Student's t-test was used to calculate statistical difference between two groups. **p < 0.01, ***p < 0.001. Source data are provided as a Source data file.

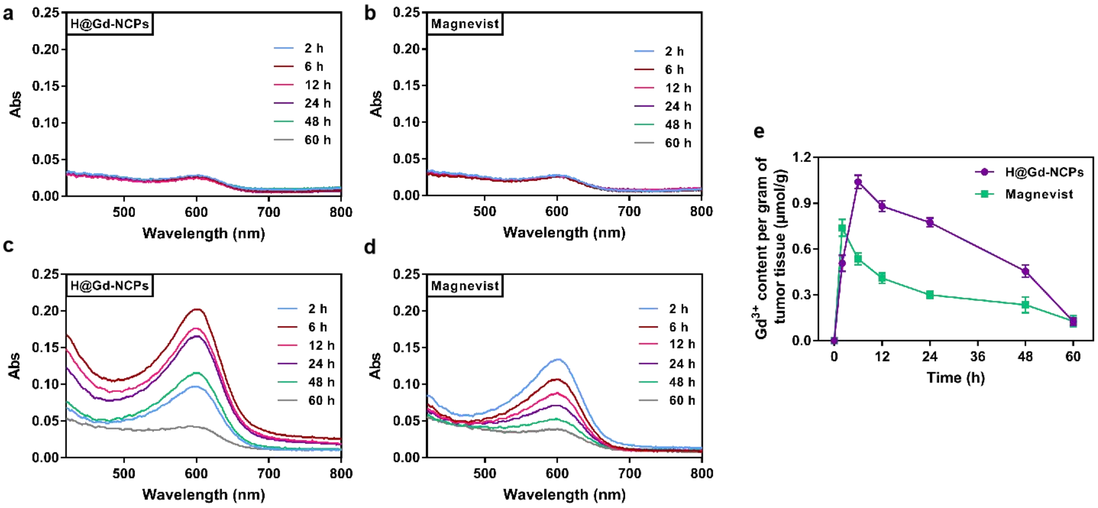

Supplementary Figure 8. Pharmacokinetic study of dynamic H@Gd-NCPs (abbreviated as H@Gd-NCPs in figures) and Magnevist. (a, b) UV spectrum of Gd^{3+} detection in H@Gd-NCPs (a) and Magnevist (b) without burning and nitrification. (c, d) UV spectrum of Gd^{3+} detection in H@Gd-NCPs (a) and Magnevist (b) after burning and nitrification. (e) The dynamic concentrations of H@Gd-NCPs or Magnevist accumulated in the tumor tissues. Data were

presented as mean ± SD (n = 3 biologically independent animals). Source data are provided as a Source data file.

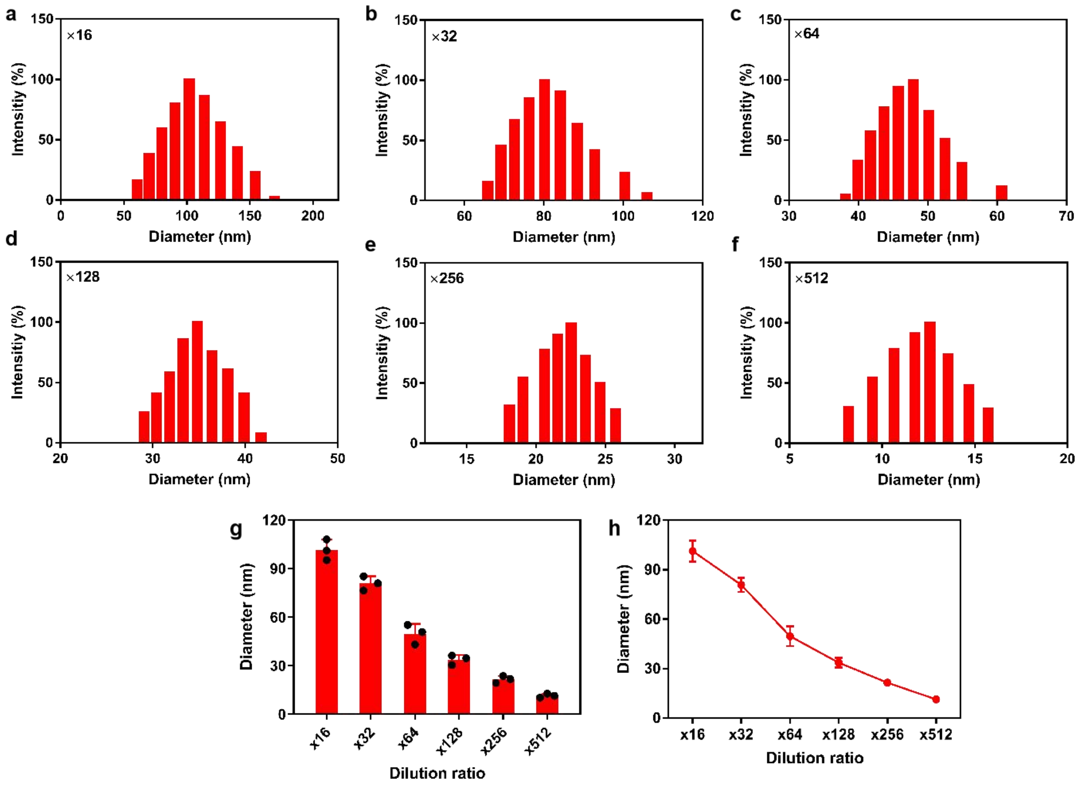

Supplementary Figure 9. Serum dilution experiments of H@Gd-NCPs in vitro. (a-f) DLS data of H@Gd-NCPs diluted 16, 32, 64, 128, 256 and 512 fold by serum at 37 °C, respectively (n = 3 biologically independent samples). (g) Histogram of H@Gd-NCPs particle size changes (n = 3 biologically independent samples). (h) Line chart of H@Gd-NCPs particle size change (n = 3 biologically independent samples). This experiment was repeated twice independently with similar results. All data were presented as mean ± SD. Source data are provided as a Source data file.

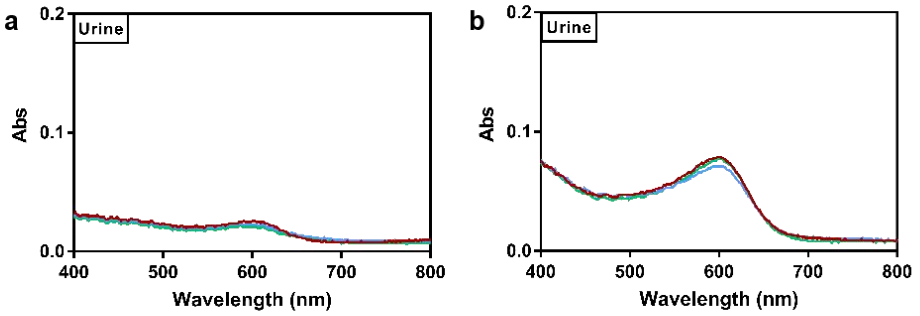

Supplementary Figure 10. Mice urine collection and analysis. (a) UV spectrum of free Gd^{3+} detection in urine without burning and nitrification. (b) DLS data of diluted urine. (n = 3 biologically independent animals). Source data are provided as a Source data file.

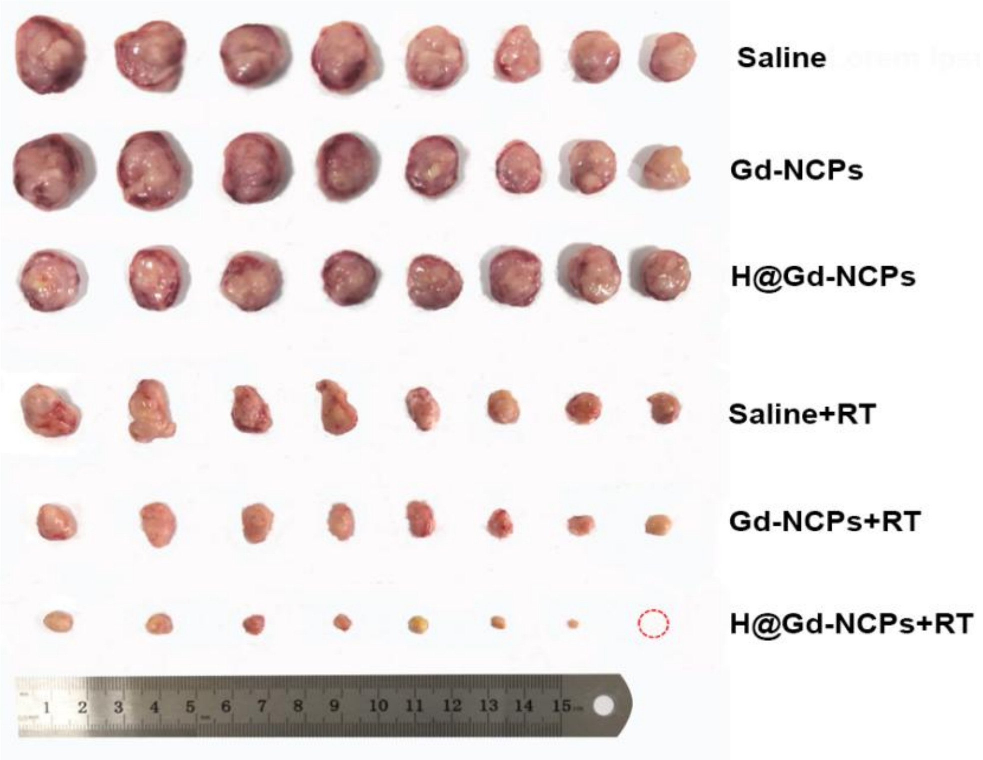

Supplementary Figure 11. Photographs of tumors (CT26) collected on day 14 (Saline, Gd-NCPs and H@Gd-NCPs groups) and day 21 (Saline+RT, Gd-NCPs+RT, H@Gd-NCPs+RT groups). H@Gd-NCPs abbreviated as H@Gd-NCPs in figures (n = 8 biologically independent animals). Detailed in vivo experimental settings please refer to the Fig. 5 legends of main text.

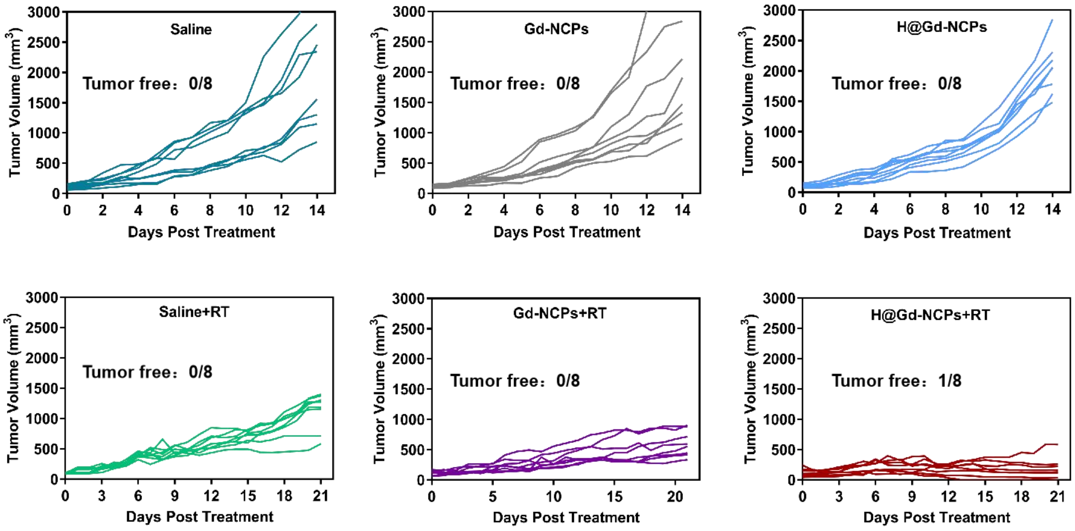

Supplementary Figure 12. Growth curves of individual tumor in different groups (n = 8 biologically independent animals). Source data are provided as a Source data file. Detailed in vivo experimental settings please refer to the Fig. 5 legends of main text.

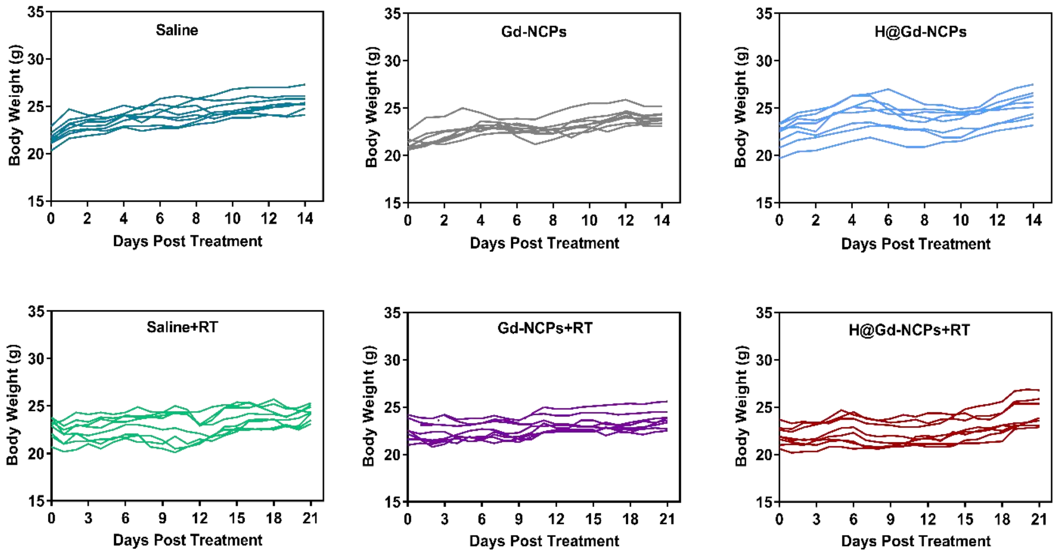

Supplementary Figure 13. Body changes of individual mouse after treatments in different groups (n = 8 biologically independent animals). Source data are provided as a Source data file. Detailed in vivo experimental settings please refer to the Fig. 5 legends of main text.

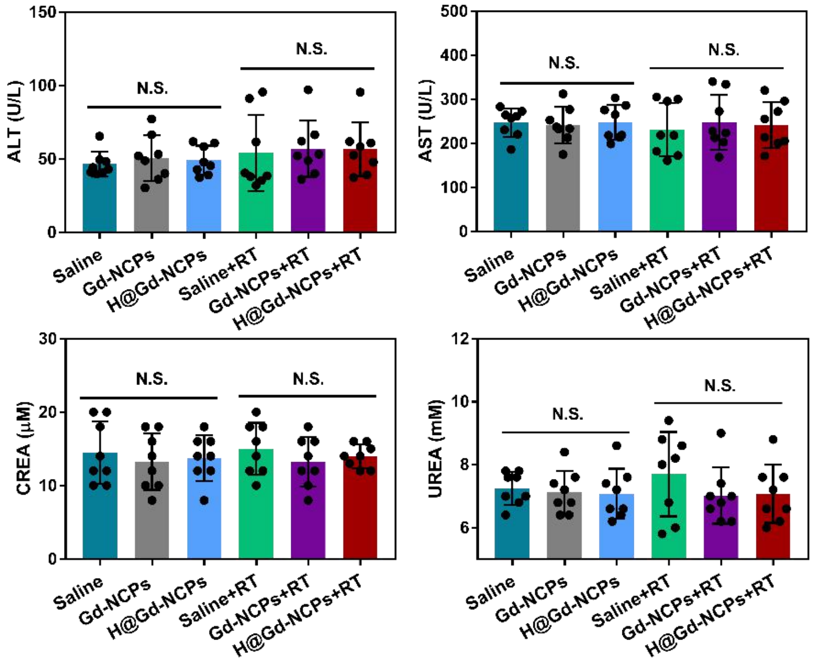

Supplementary Figure 14. Serum biochemical parameters of various treatments in CT26-bearing mice (n = 8 biologically independent samples). All data were presented as mean ± SD. Two-sided Student's *t*-test was used to calculate statistical difference between two groups. N.S. represented non-significance. Source data are provided as a Source data file. Detailed in vivo experimental settings please refer to the Fig. 5 legends of main text.

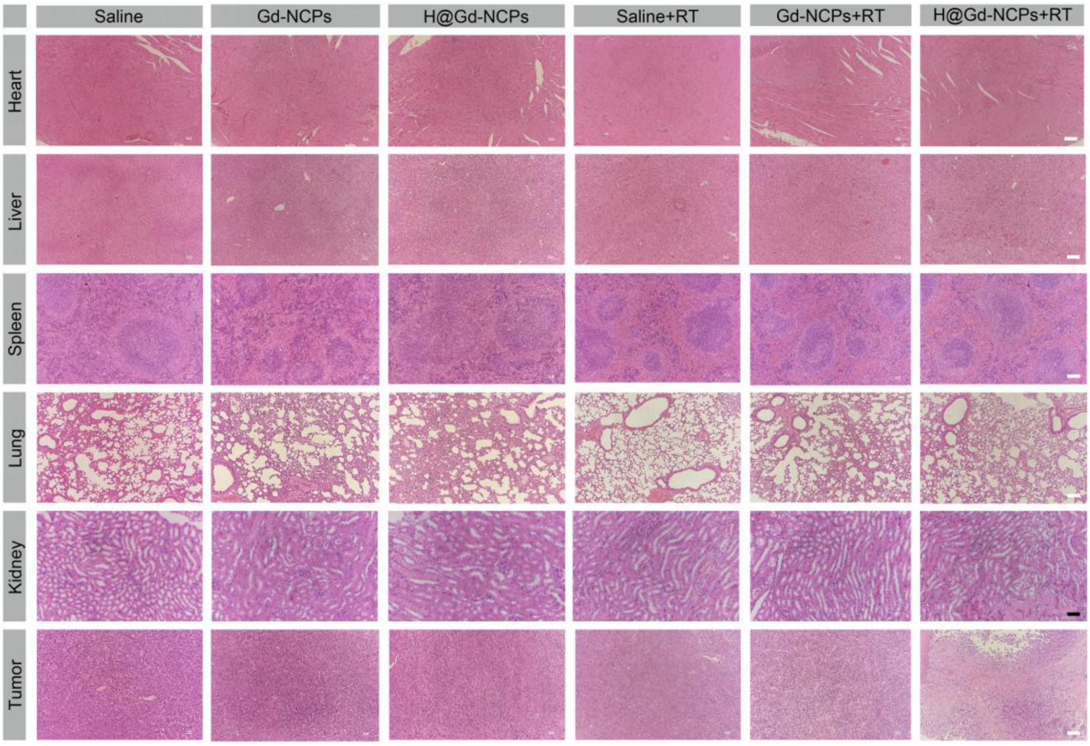

Supplementary Figure 15. H&E stain sections of Heart, Liver, Spleen, Lung, Tumor (scale bar = 100 μm) and Kidney (scale bar = 50 μm), (n = 3 biologically independent samples). This experiment was repeated twice independently with similar results. Detailed in vivo experimental settings please refer to the Fig. 5 legends of main text.

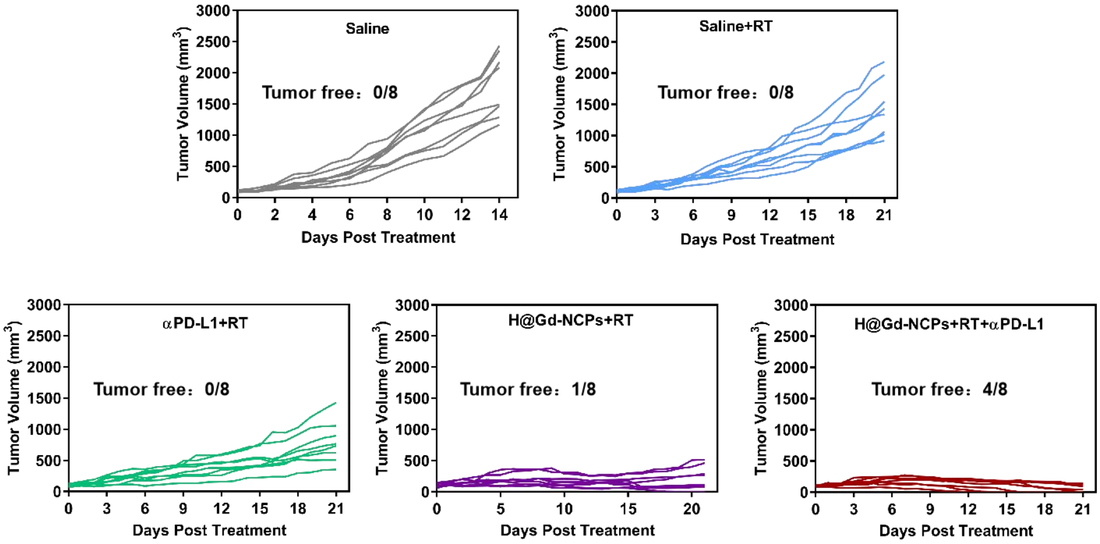

Supplementary Figure 16. Growth curves of individual primary tumor (CT26) after treatments in different groups (n = 8 biologically independent animals). Source data are provided as a Source data file. Detailed in vivo experimental settings please refer to the Fig. 7 legends of main text.

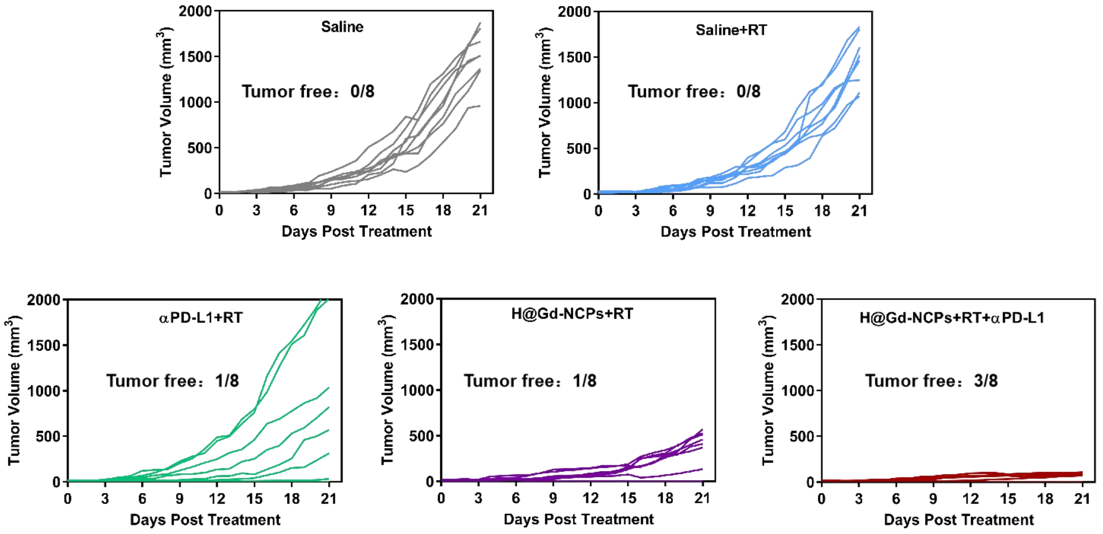

Supplementary Figure 17. Growth curves of individual distant tumor (CT26) after treatments in different groups (n = 8 biologically independent animals). Source data are provided as a Source data file. Detailed in vivo experimental settings please refer to the Fig. 7 legends of main text.

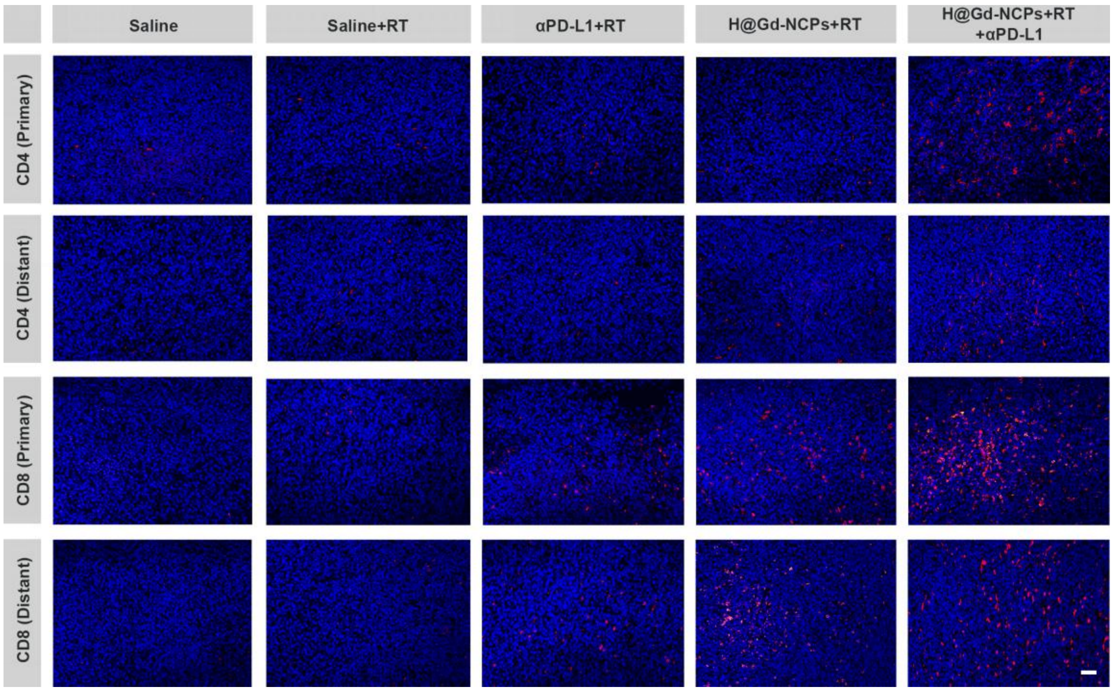

Supplementary Figure 18. Immunofluorescence staining of $CD4^+$ or $CD8^+$ T cells (red fluorescence) in primary and distant tumors (n = 3 biologically independent samples). Scale bar = 50 μm. This experiment was repeated twice independently with similar results. Detailed in vivo experimental settings please refer to the Fig. 7 legends of main text.

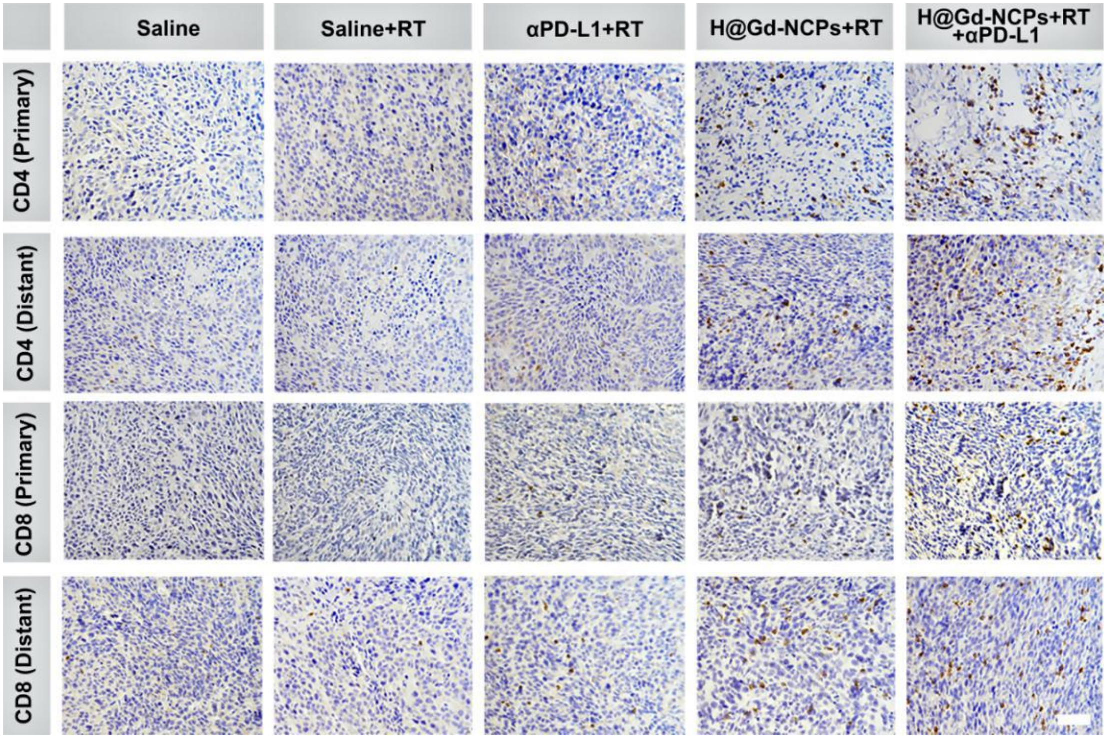

Supplementary Figure 19. Immumohistochemical staining of $CD4^{+}$ or $CD8^{+}$ T cells in primary and distant tumors (n = 3 biologically independent samples). Scale bar = 50 μm. This experiment was repeated twice independently with similar results. Detailed in vivo experimental settings please refer to the Fig. 7 legends of main text.

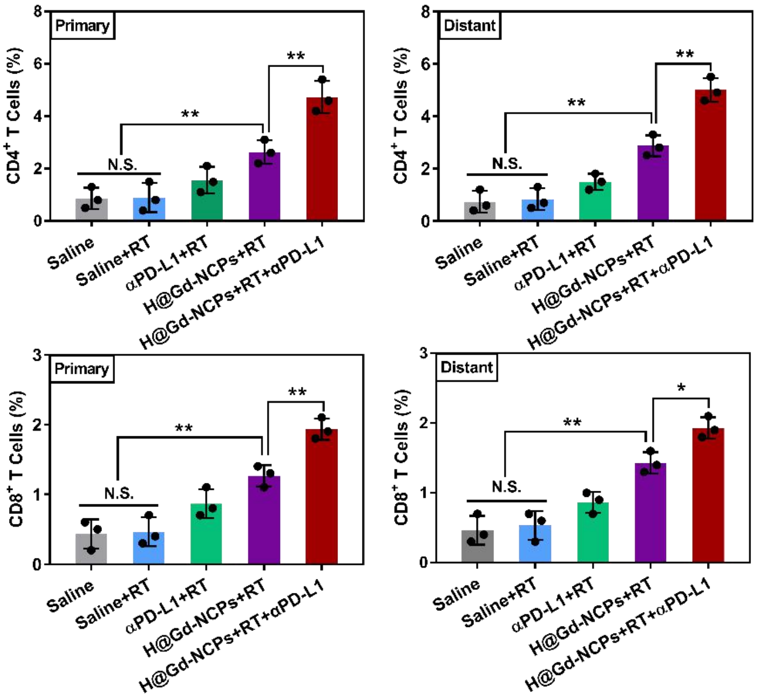

Supplementary Figure 20. Quantification of $CD4^{+}$ T and $CD8^{+}$ T cells based on IHC data from Supplementary Figure 19 (n = 3 biologically independent samples). For primary $CD4^{+}$ T cells: ***p*

= 0.0072, **p = 0.0087. For distant CD4$^+$ T cells: **p = 0.0031, **p = 0.0038. For primary CD8$^+$ T cells: **p = 0.0050, **p = 0.0059. For distant CD8$^+$ T cells: **p = 0.0029, *p = 0.0105. All data were presented as mean ± SD. Two-sided Student's t-test was used to calculate statistical difference between two groups. N.S. represented non-significance, and *p < 0.05, **p < 0.01. Source data are provided as a Source data file. Detailed in vivo experimental settings please refer to the Fig. 7 legends of main text.

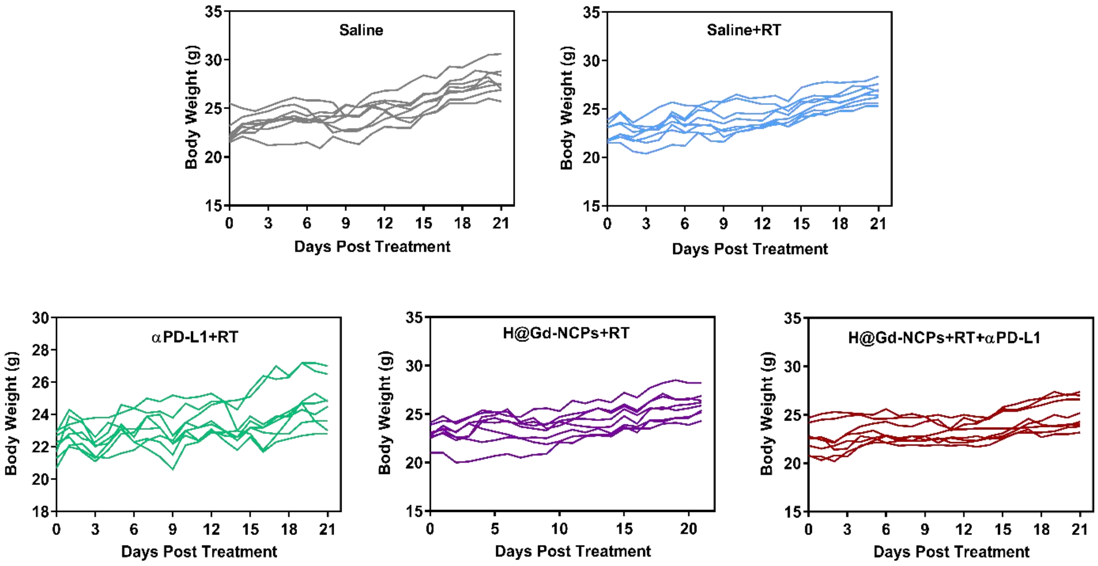

Supplementary Figure 21. Body weight change curves of individual mouse after different treatments (n = 8 biologically independent animals). Source data are provided as a Source data file. Detailed in vivo experimental settings please refer to the Fig. 7 legends of main text.

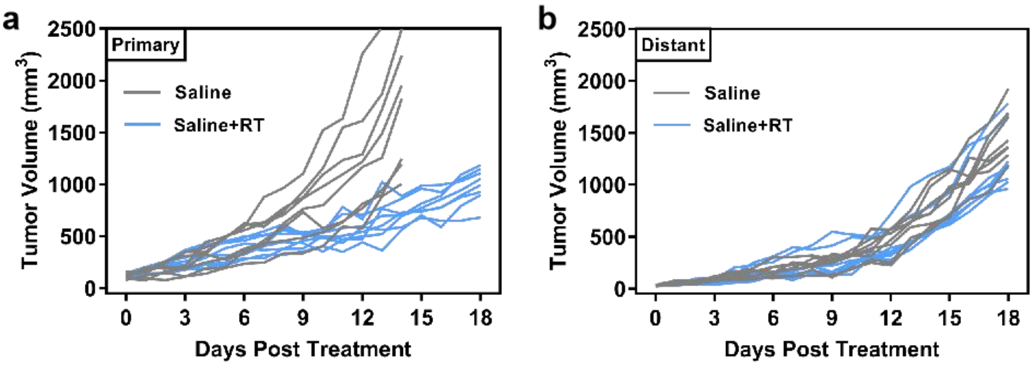

Supplementary Figure 22. (a, b) Growth curves of primary (a) and distant (b) individual tumors in the Saline and Saline+RT groups (n = 8 biologically independent animals). Source data are provided as a Source data file. Detailed in vivo experimental settings please refer to the Fig. 8 legends of main text.

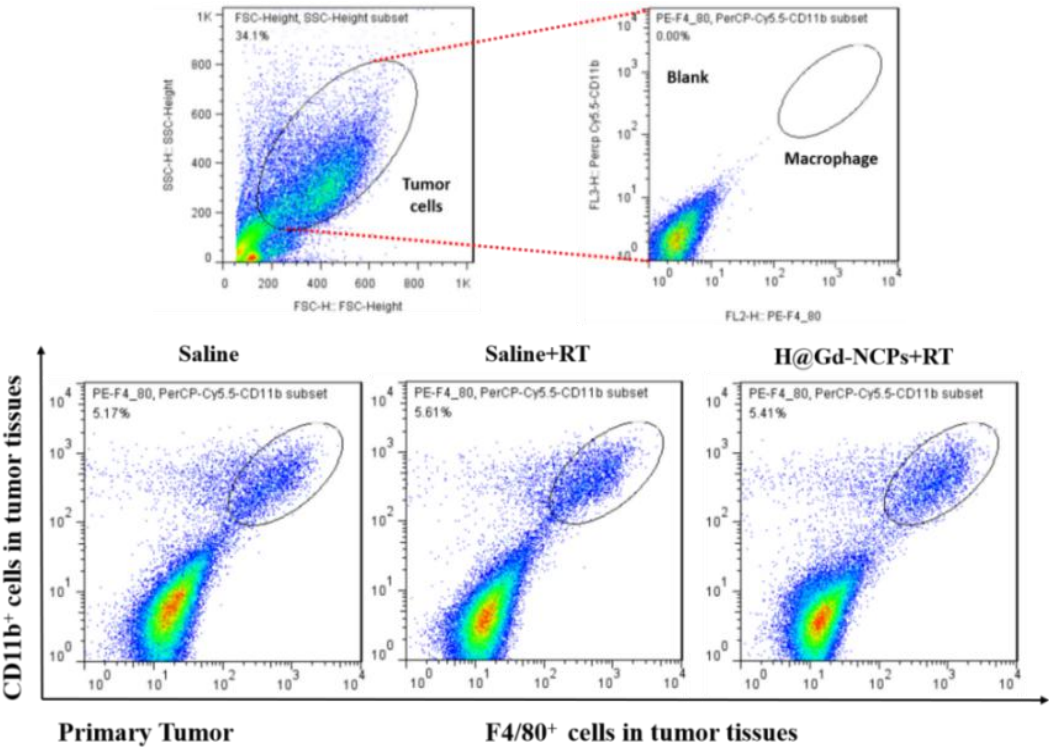

Supplementary Figure 23. F4/80$^+$ and CD11b$^+$ macrophages detected by Flow cytometry (FCM) with different treatments in CT26-bearing mice (n = 6 biologically independent animals). Detailed in vivo experimental settings please refer to the Fig. 8 legends of main text.

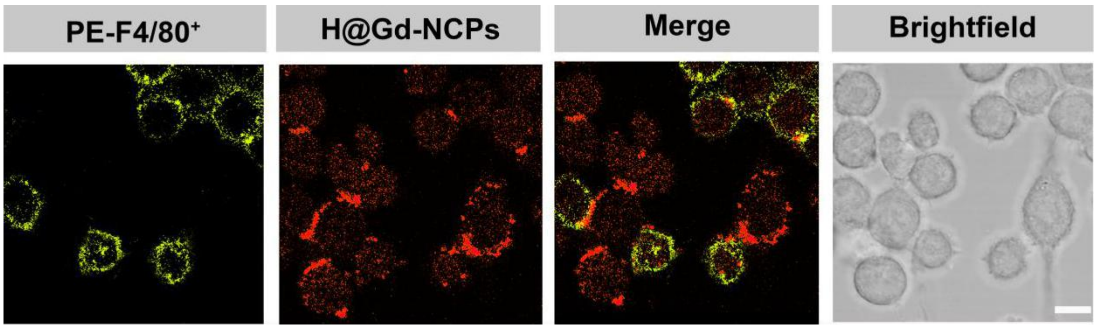

Supplementary Figure 24. Confocal laser scanning microscope (CLSM) images of co-cultured CT26 and RAW264.7 cells after treatment with PE-F4/80$^+$ and H@Gd-NCPs, respectively (n = 3 biologically independent cells). Scale bar = 10 μm. This experiment was repeated twice independently with similar results.

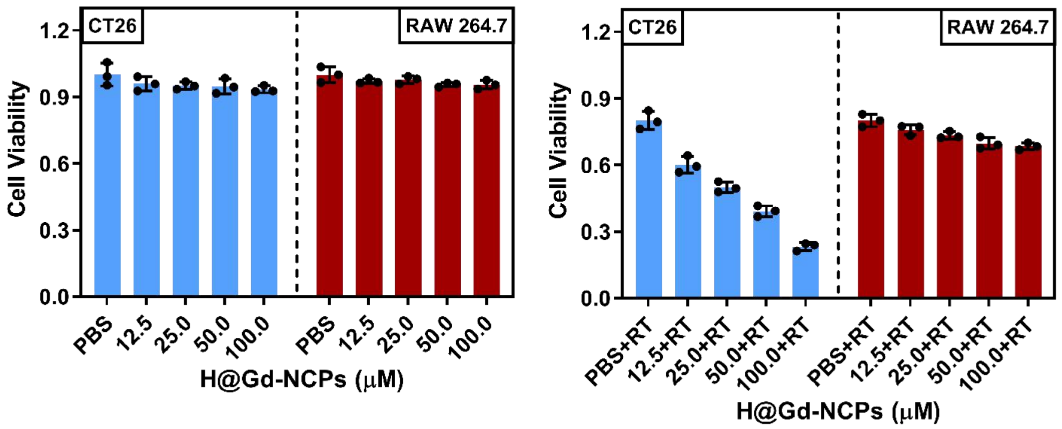

Supplementary Figure 25. The cytotoxicity of H@Gd-NCPs against CT26 and RAW264.7 cells without or with 8 Gy × 1 irradiation, respectively. [Gd^{3+}] = 0, 12.5, 25, 50, 100 μM, n = 3 biologically independent cells. This experiment was repeated twice independently with similar results. All data were presented as mean ± SD. Source data are provided as a Source data file.

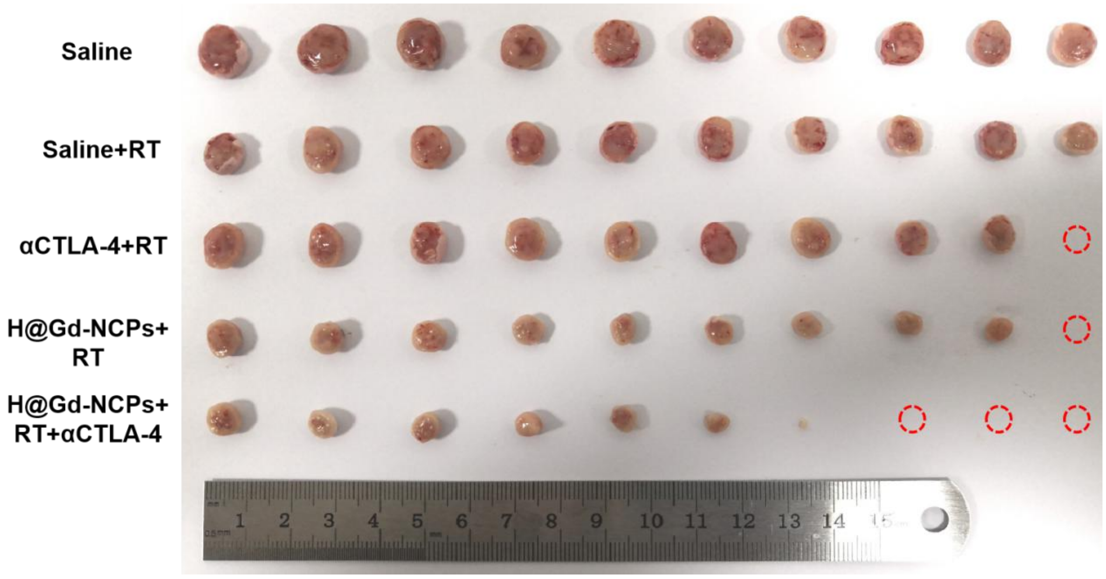

Supplementary Figure 26. Photographs of primary tumors (4T1) collected on day 15 in 4T1-bearing mice (n = 10 biologically independent animals). Detailed in vivo experimental settings please refer to the Fig. 9 legends of main text.

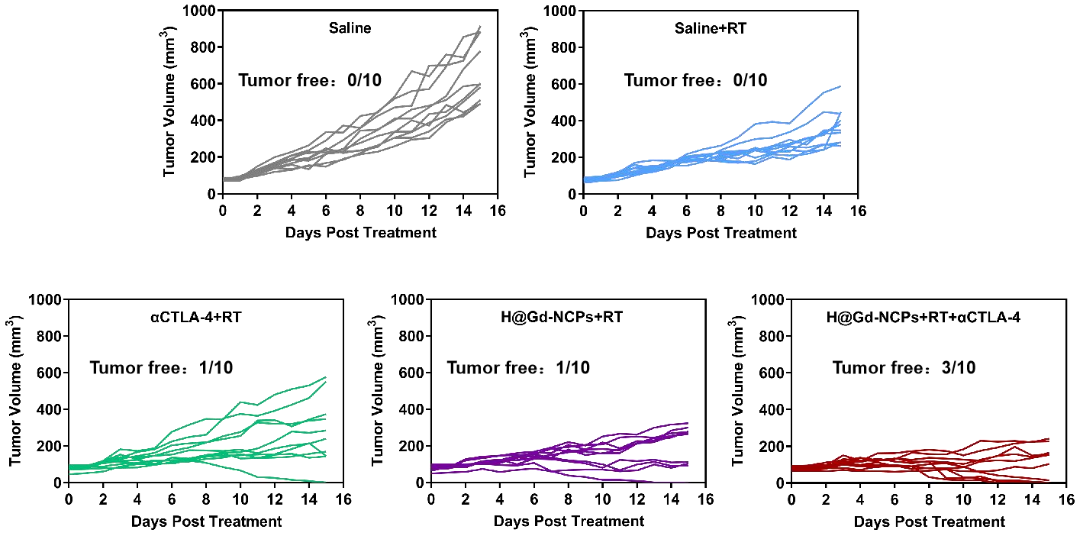

Supplementary Figure 27. Growth curves of individual 4T1 tumor after treatments in different groups (n = 10 biologically independent animals). Source data are provided as a Source data file. Detailed in vivo experimental settings please refer to the Fig. 9 legends of main text.

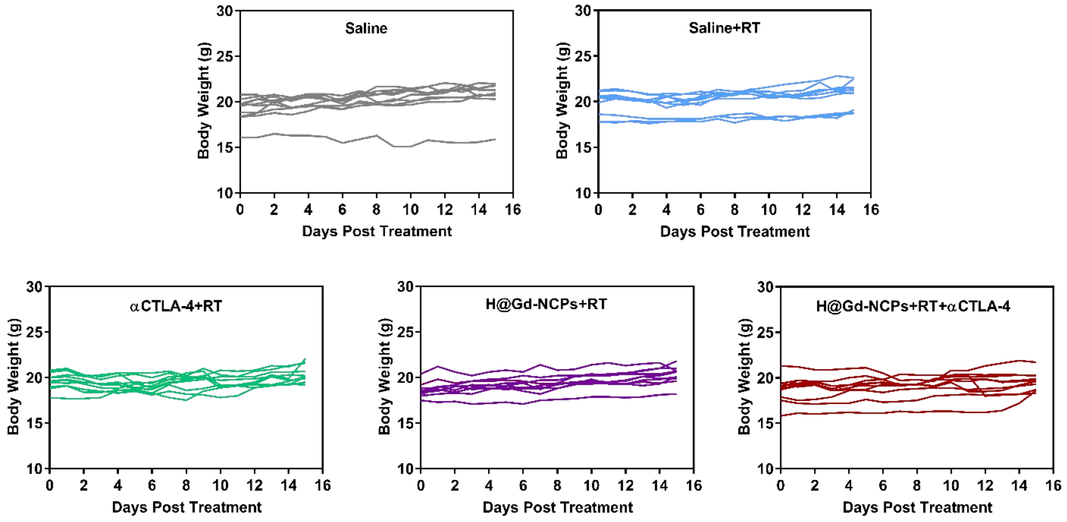

Supplementary Figure 28. Body weight change curves of individual mouse after treatments in different groups (n = 10 biologically independent animals). Source data are provided as a Source data file. Detailed in vivo experimental settings please refer to the Fig. 9 legends of main text.

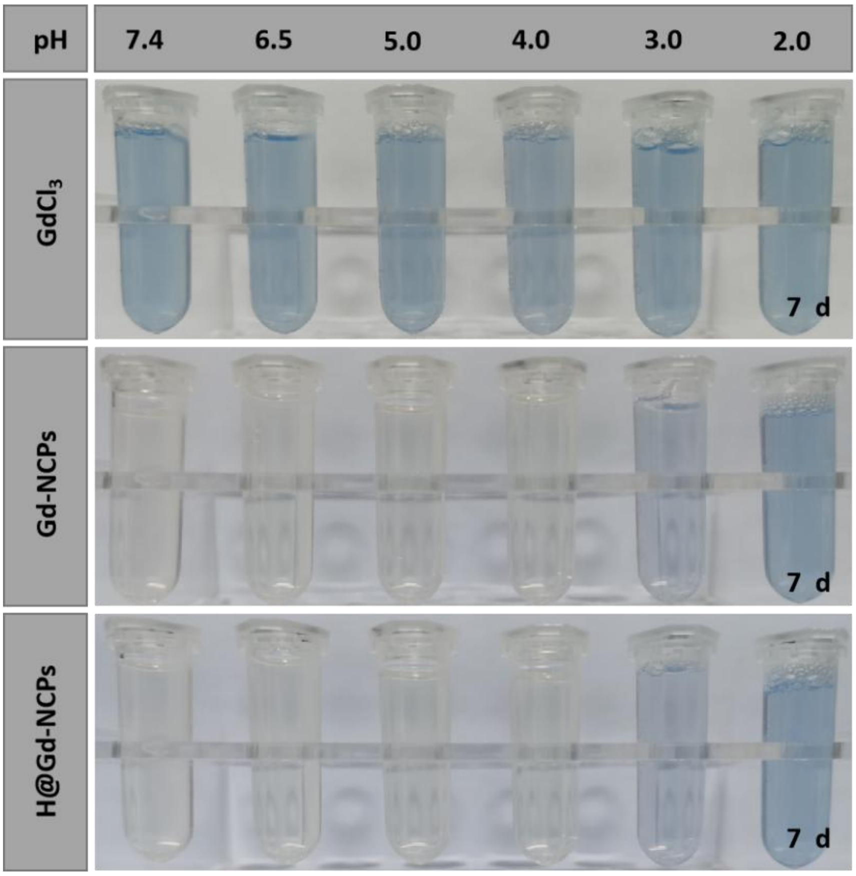

Supplementary Figure 29. Photographs of free Gd^{3+} detection by Thymolphthalein Complexon (TC) under different pH values (n = 3 biologically independent samples). This experiment was repeated twice independently with similar results.

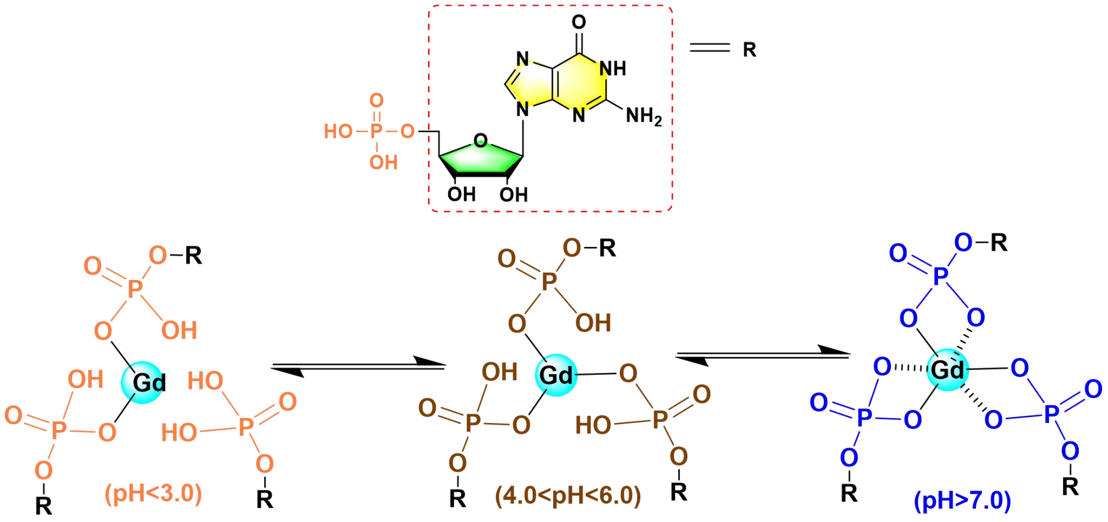

Supplementary Figure 30. Potential mechanism of the pH dependent degradation process of Gd-NCPs or H@Gd-NCPs.

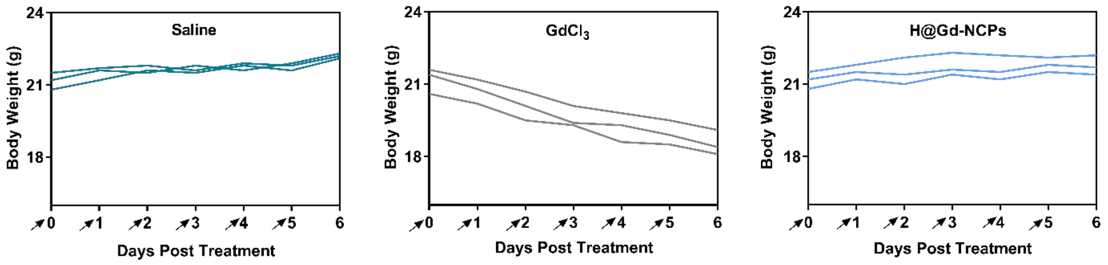

Supplementary Figure 31. Body weight change curves of individual healthy mouse after different treatments (n = 3 biologically independent animals). Source data are provided as a Source data file. Detailed in vivo experimental settings please refer to the Methods of "Acute toxicity of H@Gd-NCPs and $GdCl_3$ in vivo" in main text.

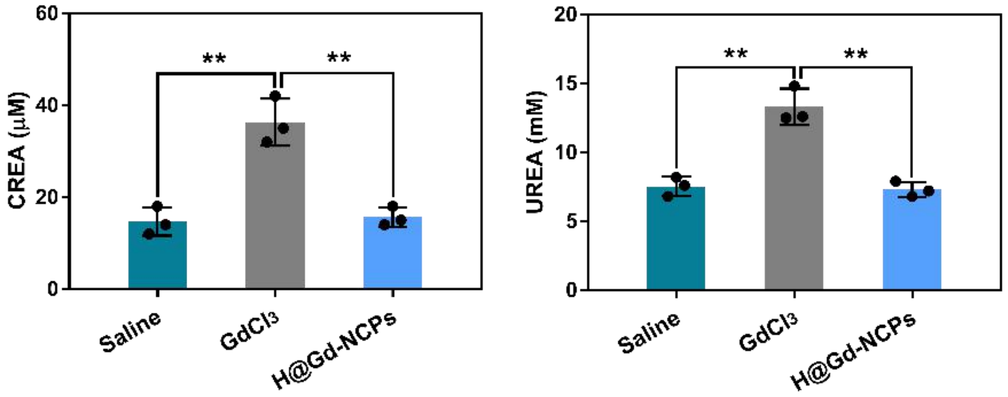

Supplementary Figure 32. Serum biochemical parameters healthy mice (n = 3 biologically independent animals) treated with Saline, $GdCl_3$ and H@Gd-NCPs. For CREA: $**p = 0.0033$, $**p = 0.0030$. For UREA: $**p = 0.0025$, $**p = 0.0018$. All data were presented as mean ± SD. Two-sided Student's *t*-test was used to calculate statistical difference between two groups. $**p < 0.01$. Source data are provided as a Source data file. Detailed in vivo experimental settings please refer to the Methods of "Acute toxicity of H@Gd-NCPs and $GdCl_3$ in vivo" in main text.

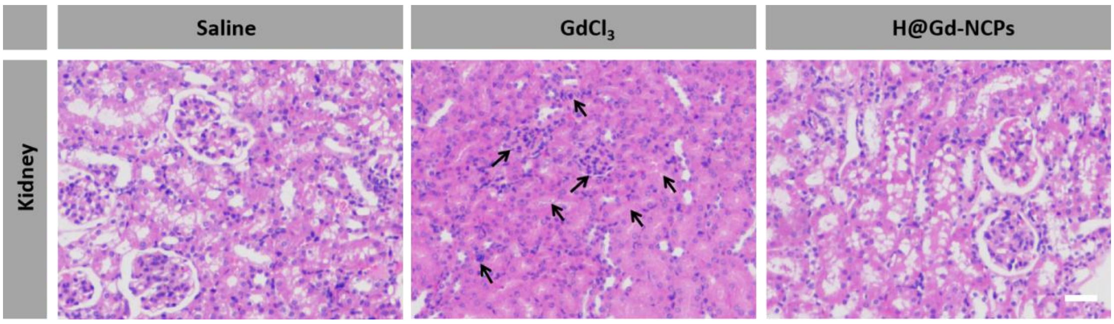

Supplementary Figure 33. H&E stain sections of kidneys treated with Saline, $GdCl_3$ and H@Gd-NCPs (n = 3 biologically independent samples). Scale bar = 50 μm. This experiment was repeated twice independently with similar results.

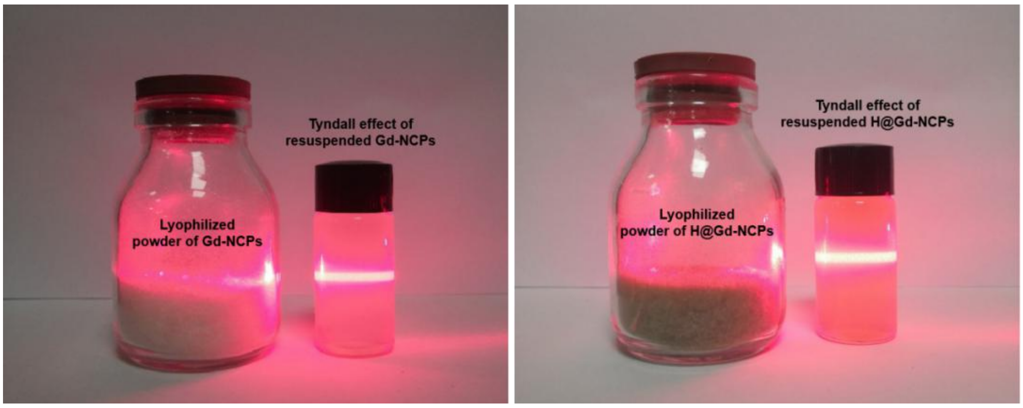

Supplementary Figure 34. Photographs of lyophilized powder of Gd-NCPs and H@Gd-NCPs, and the Tyndall effect of resuspended Gd-NCPs and H@Gd-NCPs.

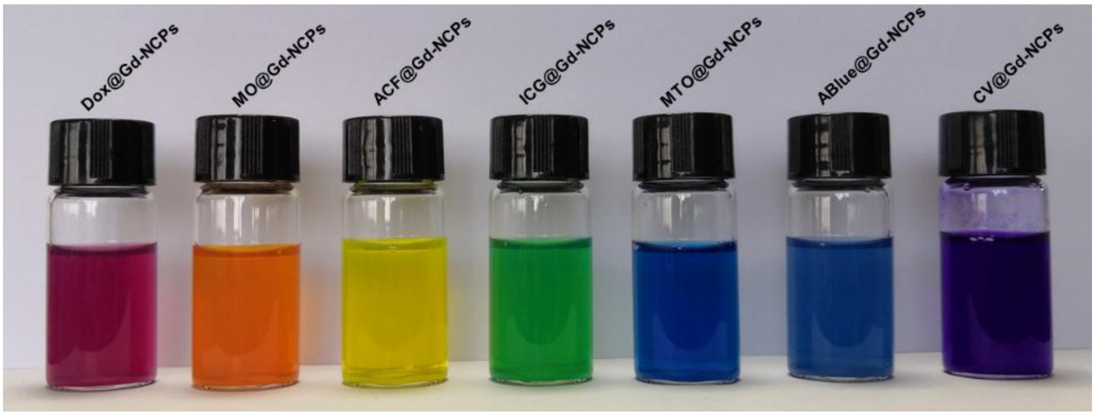

Supplementary Figure 35. Photographs of Dox@Gd-NCPs, MO@Gd-NCPs, ACF@Gd-NCPs, ICG@Gd-NCPs and MTO@Gd-NCPs, ABlue@Gd-NCPs, CV@Gd-NCPs nanoparticles. Dox (Doxorubicin), MO (Methyl Orange), ACF (Acriflavine), ICG (Indocyanine Green), MTO (Mitoxantrone), ABlue (AlcianBlue 8GX), CV (Crystal Violet).

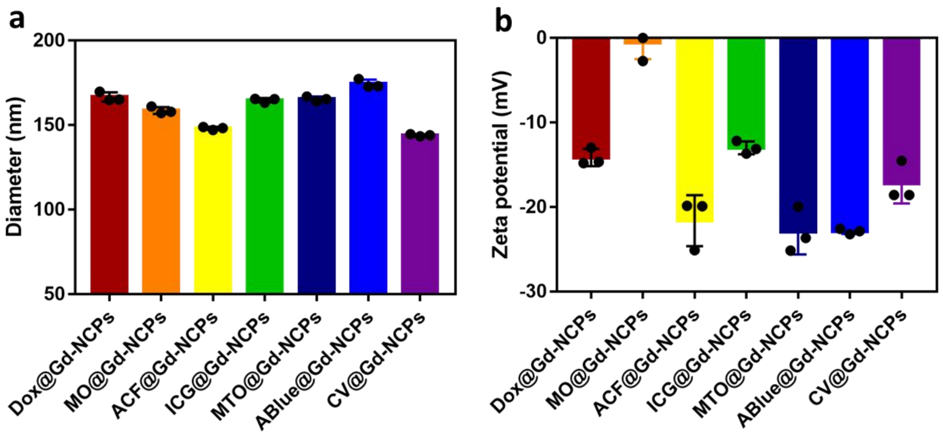

Supplementary Figure 36. Characterization of various nanoscale coordination polymers (NCPs).

Partical size (a) and zeta potential (b) of Dox@Gd-NCPs, MO@Gd-NCPs, ACF@Gd-NCPs, ICG@Gd-NCPs and MTO@Gd-NCPs, ABlue@Gd-NCPs, CV@Gd-NCPs. Dox (Doxorubicin), MO (Methyl Orange), ACF (Acriflavine), ICG (Indocyanine Green), MTO (Mitoxantrone), ABlue (AlcianBlue 8GX), CV (Crystal Violet) (n = 3 biologically independent samples). This experiment was repeated twice independently with similar results. All data were presented as mean ± SD. Source data are provided as a Source data file.

Supplementary Table 1. The calculated concentration of free Gd^{3+} *via* UV colorimetry.

**Y=0.001644X+0.02784 (SI Figure 2)**	**Free [Gd^{3+}]**		
[Gd^{3+}] = (Abs $_{605nm}$-0.02784)/0.001644	1	2	3
Gd-NCPs in deionized water	N.D.	N.D.	N.D.
H@Gd-NCPs in deionized water	N.D.	N.D.	N.D.
Gd-NCPs in serum	N.D.	N.D.	N.D.
H@Gd-NCPs in serum	N.D.	N.D.	N.D.

*N.D.: Undetectable, below [Gd^{3+}] detection limit ($<6.0\times10^{-3}$ μM).

Supplementary Table 2. The ratios of F4/80^{+} and CD11b^{+} macrophages in primary and distant tumors detected by Flow cytometry with different treatments in CT26-bearing mice. Detailed in vivo experimental settings please refer to the Fig. 8 legends of main text.

Macrophages in Primary tumor (%)	1	2	3	4	5	6	Mean ± SEM
Saline	5.17	5.59	4.96	3.62	3.20	3.04	4.26 ± 0.45
Saline+RT	5.61	4.94	4.52	4.48	3.79	3.52	4.48 ± 0.31
H@Gd-NCPs+RT	5.41	5.86	4.97	4.65	3.78	3.4	4.68 ± 0.39
