# Supplementary material for: Nanoscale coordination polymers induce immunogenic cell death by amplifying radiation therapy mediated oxidative stress
Source: Nat Commun. 2021 Jan 8;12:145. doi: 10.1038/s41467-020-20243-8 (PMC7794559; doi:10.1038/s41467-020-20243-8)
Supplement: Supplementary file 3 — Reporting Summary [file 41467_2020_20243_MOESM3_ESM.pdf]

nature research

Corresponding author(s): Yiqiao Hu, Ahu Yuan

Last updated by author(s): Nov 15, 2020

Reporting Summary

Nature Research wishes to improve the reproducibility of the work that we publish. This form provides structure for consistency and transparency in reporting. For further information on Nature Research policies, see our Editorial Policies and the Editorial Policy Checklist.

Statistics

For all statistical analyses, confirm that the following items are present in the figure legend, table legend, main text, or Methods section.

n/a	Confirmed	
☐	☒	The exact sample size (*n*) for each experimental group/condition, given as a discrete number and unit of measurement
☐	☒	A statement on whether measurements were taken from distinct samples or whether the same sample was measured repeatedly
☐	☒	The statistical test(s) used AND whether they are one- or two-sided<br>*Only common tests should be described solely by name; describe more complex techniques in the Methods section.*
☒	☐	A description of all covariates tested
☒	☐	A description of any assumptions or corrections, such as tests of normality and adjustment for multiple comparisons
☐	☒	A full description of the statistical parameters including central tendency (e.g. means) or other basic estimates (e.g. regression coefficient) AND variation (e.g. standard deviation) or associated estimates of uncertainty (e.g. confidence intervals)
☐	☒	For null hypothesis testing, the test statistic (e.g. *F*, *t*, *r*) with confidence intervals, effect sizes, degrees of freedom and *P* value noted<br>*Give P values as exact values whenever suitable.*
☒	☐	For Bayesian analysis, information on the choice of priors and Markov chain Monte Carlo settings
☒	☐	For hierarchical and complex designs, identification of the appropriate level for tests and full reporting of outcomes
☒	☐	Estimates of effect sizes (e.g. Cohen's *d*, Pearson's *r*), indicating how they were calculated

Our web collection on statistics for biologists contains articles on many of the points above.

Software and code

Policy information about availability of computer code

Data collection	Field emission scanning electron microscope (FE-SEM) images were acquired on ZEISS GeminiSEM 500. Dynamic light scattering and Zeta potential measurementsmeasurements were performed at NanoBrook 90Plus PALS Particle Size Analyzer (Brookhaven). UV-vis spectrum was recorded by a UV-VIS Spectrophotometers (UV-2450, Shimadzu). FT-IR spectrum was recorded on a Thermo Nicolet NEXUS 870 ESP FT-IR/ FT-NIR Spectrometer. X-ray photoelectron spectroscopy (XPS) was recorded by Enraf Nonius Cad 4 Turbo X-ray Diffractometer. Flow cytometry data were collected using BD FACS-Calibur and the equipped software. Tissue and tumor samples for eosin (H&E) staining were observed using a Nikon Eclipse Ti Fluorescence Microscope. Magnetic resonance imaging (MRI) measurements were performed on MR scanner (Biospec 7T/20 USR, Germany). Tumor size and weight of tumor-bearing mice were recorded with Microsoft Office 2019.
Data analysis	GraphPad Prism Version 7.0; ImageJ Version 1.52v; FlowJo Version 7.6.1; Sante MRI Viewer 3.0; NIS-Elements Viewer 5.21.00

For manuscripts utilizing custom algorithms or software that are central to the research but not yet described in published literature, software must be made available to editors and reviewers. We strongly encourage code deposition in a community repository (e.g. GitHub). See the Nature Research guidelines for submitting code & software for further information.

Data

Policy information about availability of data

All manuscripts must include a data availability statement. This statement should provide the following information, where applicable:

- Accession codes, unique identifiers, or web links for publicly available datasets
- A list of figures that have associated raw data
- A description of any restrictions on data availability

The source data underlying Figures 2b-i, 3c-f, 4b-c, 4f-h, 5a-c, 5e-g, 6b-e, 7a-l, 8a-i, 9b-f, 9h-i and Supplementary Figures 2a-d, 3-5, 7-10, 12-14, 16-17, 20-22, 25, 27-28, 31-32, 36 are available as a Source Data file. The remaining data are available within the Article, Supplementary Information or available from the authors

upon request. A reporting summary for this study is available as a Supporting Information file. Chemical states of elements are assigned based on the National Institute of Standards and Technology (NIST) XPS Database (DOI: http://dx.doi.org/10.18434/T4T88K).

Field-specific reporting

Please select the one below that is the best fit for your research. If you are not sure, read the appropriate sections before making your selection.

[x] Life sciences [ ] Behavioural & social sciences [ ] Ecological, evolutionary & environmental sciences

For a reference copy of the document with all sections, see nature.com/documents/nr-reporting-summary-flat.pdf

Life sciences study design

All studies must disclose on these points even when the disclosure is negative.

Sample size	Sample sizes were assigned based upon historical practices, in line with recent relevant publications, for instance, Lu, K. et al. Low-dose X-ray radiotherapy-radiodynamic therapy via nanoscale metal-organic frameworks enhances checkpoint blockade immunotherapy. Nat. Biomed. Eng. 2, 600-610 (2018).
Data exclusions	No data were excluded from analysis.
Replication	All experiments were replicated independently 2-3 times, as indicated in figure legends.
Randomization	Cell samples were randomly allocated to treatment by random division into multi-well plates. Animals were randomized by random number.
Blinding	Investigators were blinded to group allocation during data collection and analysis for both in vitro and in vivo experiments.

Reporting for specific materials, systems and methods

We require information from authors about some types of materials, experimental systems and methods used in many studies. Here, indicate whether each material, system or method listed is relevant to your study. If you are not sure if a list item applies to your research, read the appropriate section before selecting a response.

Materials & experimental systems

n/a	Involved in the study	
[ ]	[x]	Antibodies
[ ]	[x]	Eukaryotic cell lines
[x]	[ ]	Palaeontology and archaeology
[ ]	[x]	Animals and other organisms
[x]	[ ]	Human research participants
[x]	[ ]	Clinical data
[x]	[ ]	Dual use research of concern

Methods

n/a	Involved in the study	
[x]	[ ]	ChIP-seq
[ ]	[x]	Flow cytometry
[x]	[ ]	MRI-based neuroimaging

Antibodies

Antibodies used

Anti-HMGB1 antibody, Cat# ab18256, diluted 1:300 with 3% BSA, Abcam, USA.
Anti-Calreticulin antibody [EPR3924] - ER Marker (Alexa Fluor® 488), Cat# ab196158, diluted 1:500 with 3% BSA, Abcam, USA.
Anti-gamma H2A.χ (phospho S139) antibody [9F3], Cat# ab26350, diluted 1:200 with 3% BSA, Abcam, USA.
Goat Anti-Mouse IgG H&L (Alexa Fluor® 488), Cat# ab150113, diluted 1:200 with 3% BSA, Abcam, USA.
Anti-Ki67 antibody, Cat# ab15580, diluted 1:500 with 3% BSA, Abcam, USA.
Goat Anti-Mouse IgG H&L (HRP), Cat# ab6789, diluted 1:300 with 3% BSA, Abcam, USA.
TUNEL Assay Kit-BrdU-Red, Cat# ab66110, diluted 1:200 with 3% BSA, Abcam, USA.
Anti-CD4 antibody [EPR19514], Cat# ab183685, diluted 1:400 with 3% BSA, Abcam, USA.
Anti-CD8 alpha antibody [EPR21769], Cat# ab217344, diluted 1:400 with 3% BSA, Abcam, USA.
Goat Anti-Mouse IgG H&L (Cy3 ®) preadsorbed, Cat# ab97035, diluted 1:300 with 3% BSA, Abcam, USA.
FITC anti-mouse CD11c Antibody [N418], Cat# 117306, 0.25 μg per million cells in 100 μL volume, BioLegend, USA.
APC anti-mouse CD80 Antibody [16-10A1], Cat# 104713, 1.0 μg per million cells in 100 μL volume, BioLegend, USA.
PE anti-mouse CD86 Antibody [GL-1], Cat# 105007, 0.25 μg per million cells in 100 μL volume, BioLegend, USA.
APC anti-mouse CD3 Antibody [17A2], Cat# 100236, 0.5 μg per million cells in 100 μL volume, BioLegend, USA.
PE anti-mouse CD4 Antibody [GK1.5], Cat# 100408, 0.25 μg per million cells in 100 μL volume, BioLegend, USA.
FITC anti-mouse CD8a Antibody [53-6.7], Cat# 100706, 1.0 μg per million cells in 100 μL volume, BioLegend, USA.
PE anti-mouse CD3 Antibody [17A2], Cat# 100205, 0.5 μg per million cells in 100 μL volume, BioLegend, USA.
APC anti-mouse/human CD44 Antibody [IM7], Cat# 103011, 0.25 μg per million cells in 100 μL volume, BioLegend, USA.
PerCP/Cyanine5.5 anti-mouse CD62L Antibody [MEL-14], Cat# 104432, 0.25 μg per million cells in 100 μL volume, BioLegend, USA.

	PE anti-mouse F4/80 Antibody [BM8], Cat# 123110, 1.0 µg per million cells in 100 µL volume, BioLegend, USA.<br>PerCP/Cyanine5.5 anti-mouse/human CD11b Antibody [M1-70], Cat# 101227, 0.25 µg per million cells in 100 µL volume, BioLegend, USA.<br>Ultra-LEAF™ Purified anti-mouse CD8a Antibody [53-6.7], Cat# 100764, BioLegend, USA.<br>In Vivo MAb anti-mouse PD-L1(B7-H1) [Clone: 10F.9G2], Cat# BE0101, BioXcell, USA.<br>In Vivo MAb anti-mouse CTLA-4 (CD152) [Clone: UC10-4F10-11], Cat# BE0032, BioXcell, USA.
Validation	All antibodies were verified by the supplier and each lot has been quality tested.<br>https://www.abcam.com/hmgb1-antibody-ab18256.html<br>https://www.abcam.com/calreticulin-antibody-epr3924-er-marker-alexa-fluor-488-ab196158.html<br>https://www.abcam.com/gamma-h2ax-phospho-s139-antibody-9f3-ab26350.html<br>https://www.abcam.com/goat-mouse-igg-hl-alexa-fluor-488-ab150113.html<br>https://www.abcam.com/ki67-antibody-ab15580.html<br>https://www.abcam.com/goat-mouse-igg-hl-hrp-ab6789.html<br>https://www.abcam.com/tunel-assay-kit-brdu-red-ab66110.html<br>https://www.abcam.cn/cd4-antibody-epr19514-ab183685.html<br>https://www.abcam.com/cd8-alpha-antibody-epr21769-ab217344.html<br>https://www.abcam.com/goat-mouse-igg-hl-cy3--preadsorbed-ab97035.html<br>https://www.biolegend.com/en-gb/products/fitc-anti-mouse-cd11c-antibody-1815<br>https://www.biolegend.com/en-gb/products/apc-anti-mouse-cd80-antibody-2340<br>https://www.biolegend.com/en-gb/products/pe-anti-mouse-cd86-antibody-256<br>https://www.biolegend.com/en-gb/products/apc-anti-mouse-cd3-antibody-8055<br>https://www.biolegend.com/en-gb/products/pe-anti-mouse-cd4-antibody-250<br>https://www.biolegend.com/en-gb/products/fitc-anti-mouse-cd8a-antibody-153<br>https://www.biolegend.com/en-gb/products/pe-anti-mouse-cd3-antibody-47<br>https://www.biolegend.com/en-gb/products/apc-anti-mouse-human-cd44-antibody-312<br>https://www.biolegend.com/en-gb/products/percp-cyanine5-5-anti-mouse-cd62l-antibody-4272<br>https://www.biolegend.com/en-gb/products/pe-anti-mouse-f4-80-antibody-4068<br>https://www.biolegend.com/en-gb/products/percp-cyanine5-5-anti-mouse-human-cd11b-antibody-4257<br>https://www.biolegend.com/en-gb/products/ultra-leaf-purified-anti-mouse-cd8a-antibody-7731<br>https://bxcell.com/product/m-pdl-1/<br>https://bxcell.com/product/m-cd152-m-ctla-4-2/

Eukaryotic cell lines

Policy information about cell lines

Cell line source(s)	The mouse CT26 colorectal cancer cells and 4T1 breast cancer cells were purchased from China Type Culture Collection, supplied by the American Type Culture Collectio.
Authentication	All the cells were purchased from China Type Culture Collection (CTCC) obtained from the American Type Culture Collection (ATCC). No authentication was done by ourself.
Mycoplasma contamination	Cells were routinely tested negative for mycoplasma contamination using MycoSET Mycoplasma real-time PCR detection Kit.
Commonly misidentified lines (See ICLAC register)	No misidentified lines.

Animals and other organisms

Policy information about studies involving animals; ARRIVE guidelines recommended for reporting animal research

Laboratory animals	BALB/c mice (Male, 5 weeks) for the construction of CT26-bearing mice and BALB/c mice (Female, 5 weeks) for the construction of 4T1-bearing mice. The animals were hosted in equipped animal facility with temperature at 68-79 F and humidity at 30%-70%, under the same dark/light cycle (12:12).
Wild animals	The study did not involve wild animals.
Field-collected samples	The study did not involve any samples collected from field.
Ethics oversight	All the animals were obtained from Yangzhou university medical center (Yangzhou, China) and received care in accordance with Institution Animal Care and Use Committee (IACUC) of Nanjing University.

Note that full information on the approval of the study protocol must also be provided in the manuscript.

Flow Cytometry

Plots

Confirm that:

☒ The axis labels state the marker and fluorochrome used (e.g. CD4-FITC).

☒ The axis scales are clearly visible. Include numbers along axes only for bottom left plot of group (a 'group' is an analysis of identical markers).

☒ All plots are contour plots with outliers or pseudocolor plots.

☒ A numerical value for number of cells or percentage (with statistics) is provided.

Methodology

Sample preparation	Cell samples were collected from primary and distant murine subcutaneous tumor tissues, digested with collagenase/dispase solution, and passed through single-cell filters
Instrument	FACS data was collected using BD FACS Calibur flow cytometer
Software	FACS data was collected using BD FACS Calibur and analyzed using Flow Jo 7.6.1
Cell population abundance	FACS analysis was performed on each sample to a total cell number between 50,000 to 1,000,00 events with a threshold of 10,000 to increase quality of samples per event. Each gated population was sorted so that at least 500 cells for the furthest gated cell population was recorded to obtain satisfactory percentage of the cell population. FACS quality was also ensured using compensation controls and FMO controls to verify that observed and gated populations were accurate and distinct
Gating strategy	All of the flow cytometry experiments were adopted with this sample treatment method and gating strategy. After incubated with various antibodies, cells were fixed by 4% paraformaldehyde and then analysed via flow cytometry. During the the running process, Forward Scatter (FSC) and Side Scatter (SSC) dot maps were established, the voltage was adjusted to ensure that all the events were within the visible range of the dot maps. Then, the events with appropriate FSC (200-600) and SSC (200-600) were gated and collected. Those events with low FSC/low SSC and low FSC/high SSC were abandoned, which mainly represented cell debris and air bubbles. Followed by cell type specific gating using fluorescently labeled antibodies. Dendritic cells (CD80+ and CD86+ gated on CD11c+); CD4+ T cells (CD3+ and CD4+); CD8+ T cells (CD3+ and CD8+); Effector memory T cells (TEM, CD62L– CD44+ gated on CD3+ CD8+); Macrophages (F4/80+ and CD11b+).

☒ Tick this box to confirm that a figure exemplifying the gating strategy is provided in the Supplementary Information.
